# To know or not to know? Curiosity and the value of prospective information in animals

**DOI:** 10.3758/s13420-024-00647-y

**Published:** 2024-10-16

**Authors:** Victor Ajuwon, Tiago Monteiro, Alexandra K. Schnell, Nicola S. Clayton

**Affiliations:** 1 Department of Psychology, University of Cambridge, Cambridge, UK; 2 William James Centre for Research, University of Aveiro, Aveiro, Portugal; 3 Domestication Lab, Konrad Lorenz Institute of Ethology, Department of Interdisciplinary Life Sciences, University of Veterinary Medicine Vienna, Vienna, Austria

**Keywords:** Comparative cognition, Memory, Operant conditioning, Metacognition

## Abstract

Humans and other animals often seek instrumental information to strategically improve their decisions in the present. Our curiosity also leads us to acquire non-instrumental information that is not immediately useful but can be encoded in memory and stored for use in the future by means of episodic recall. Despite its adaptive benefits and central role in human cognition, questions remain about the cognitive mechanisms and evolutionary origins that underpin curiosity. Here, we comparatively review recent empirical studies that some authors have suggested reflects curiosity in nonhuman animals. We focus on findings from laboratory tasks in which individuals can choose to gain advanced information about uncertain future outcomes, even though the information cannot be used to increase future rewards and is often costly. We explore the prevalence of preferences in these tasks across animals, discuss the theoretical advances that they have promoted, and outline some limitations in contemporary research. We also discuss several features of human curiosity that can guide future empirical research aimed at characterising and understanding curiosity in animals. Though the prevalence of curiosity in animals is actively debated, we surmise that investigating behavioural candidates for curiosity-motivated behaviour in a broader range of species and contexts, should help promote theoretical advances in our understanding of cognitive principles and evolutionary pressures that support curiosity-driven behaviour.

## The value of information

Uncertainty is a ubiquitous challenge for living organisms. To deal with this, across contexts diverse as foraging, predator avoidance, and mate selection, animals have evolved a range of strategies to acquire biologically relevant information (Gottlieb et al., 2013). For example, in laboratory foraging tasks, individuals can learn to acquire information to improve reward outcomes (Foley et al., 2017; Gottlieb et al., 2014; Kobayashi & Hsu, 2019) and can forgo rewards in the short term to seek information that will enhance their long-term gains—a challenging trade-off labelled the exploration-exploitation dilemma (Berger-Tal et al., 2014; Cohen et al., 2007; Krebs et al., 1978; see also Schnell et al. 2021b, Schnell et al., 2022, for the links between patience for future rewards and cognitive flexibility). In these examples, information has extrinsic value, and information-seeking is referred to as instrumental because it improves the efficiency of goal-directed actions, providing immediate, direct benefits in the form of primary reinforcement. Such information-seeking is consistent with normative models of value-based decision-making that emphasise the adaptive benefits of reward-maximisation (Stephens & Krebs, 1986).

Conversely, in other contexts, information-seeking appears to be a means unto itself reflecting *intrinsic* value that agents attribute to information (Anselme, 2023; Gottlieb & Oudeyer, 2018). For example, we read simply to learn the fate of our favourite fictional characters, travel leisurely for new experiences, and some of us dedicate entire careers to the generation of basic scientific knowledge. Information-seeking behaviour of this kind is often referred to as non-instrumental (Bennett et al., 2016; Bromberg-Martin & Monosov, 2020; Gottlieb & Oudeyer, 2018; Monosov, 2024) and has recently been emphasised as a key feature of curiosity (Gottlieb et al., 2020; Gottlieb & Oudeyer, 2018; Kidd & Hayden, 2015). Formally, curiosity is difficult to define, but it can broadly be characterised as (1) the drive to explore novel objects, often studied by examining attention and gaze (Gottlieb et al., 2014; Monosov, 2024), and (2) an intrinsic motivation to acquire information in the absence of instrumental incentives, with the aim of uncertainty reduction (Berlyne, 1960; Bromberg-Martin & Monosov, 2020), or the filling of ‘information gaps’ (Loewenstein, 1994). Though non-instrumental information-seeking does not result in immediate, tangible benefits, this tendency likely has long-term benefits, enabling decision-makers to enrich their representation of the world and enhance future problem-solving.

There is little doubt about the significant role that curiosity has in motivating and affecting human attention, decision-making, and memory (Gottlieb et al., 2014; Gruber & Ranganath, 2019; Loewenstein & Molnar, 2018; Sharot & Sunstein, 2020), yet open questions remain about the cognitive mechanisms, neural substrates, and evolutionary origins that underpin curiosity. The comparative approach provides a powerful opportunity to draw inferences about the cognitive principles and evolutionary pressures that support curiosity driven behaviour.

Here, taking a comparative approach, we critically review empirical findings that some authors interpret to suggest nonhuman animals show curiosity or ‘curiosity-like’ behaviour. We focus primarily on findings from a decision-making paradigm variously referred to as ‘suboptimal choice’, ‘paradoxical choice’, and ‘non-instrumental choice’ (e.g., Ajuwon et al., 2023; Vasconcelos et al., 2015), in which animals can choose to observe predictive cues about forthcoming reward outcomes but cannot use the information to increase tangible rewards available to them. We discuss the theoretical implications interpretations resulting from these experiments, before highlighting avenues for future research aimed at exploring the evolution and prevalence of curiosity in animals.

## Empirical approaches to animal curiosity

The question of whether animals are curious has attracted the interest of psychologists such as Pavlov (1927) and Skinner (1938), but over decades it has been a difficult one to answer. This is in part because psychologists have lacked a unified definition of curiosity (Modirshanechi et al., 2023), and perhaps because our notion of curiosity is tied to our subjective experience of it—a phenomenon that can be investigated via self-report in humans (e.g., Gruber et al., 2014; Kang et al., 2009; Lau et al., 2020) but is impossible to examine in other animals (an issue we return to later on that is also mirrored in the comparative study of episodic memory started by Clayton & Dickinson, 1998; see Clayton et al., 2003; Jelbert & Clayton, 2017).

Nonetheless, over the years studies across a range of species have investigated behaviours that promote novel experiences and the acquisition of non-instrumental information—behaviour that is apparently motivated by curiosity. In novel object paradigms, subjects are presented with novel or surprising stimuli that can evoke rapid, reactive sensory inspection and exploration (Berlyne, 1950, 1970; Jaegle et al., 2019; Monosov, 2024; Sokolov, 1963). Orienting responses can involve physiological changes, such as changes in pupil diameter and respiration (Nieuwenhuis et al., 2011), but habituate rapidly, highlighting that they reflect a general, nonassociative form of learning. Differences in the intensity of orienting responses and the rate of subsequent habituation towards novel objects have been regarded as quantitative measures of curiosity (Byrne, 2013; Glickman & Sroges, 1966).

As well as reactions to presented novel objects, researchers also investigate how agents actively seek novel stimuli (Jaegle et al., 2019; Monosov et al., 2022). Using a gaze-shift paradigm, a recent study in monkeys showed that subjects learnt to generate novel visual stimuli independently of increases in instrumental rewards, preferring to shift their gaze so as to produce novel rather than familiar stimuli (Ogasawara et al., 2022). Furthermore, Ahmadlou et al. (2021) recently used a free-access choice paradigm to investigate novelty-seeking, showing that optogenetic stimulation can induce preferences for interaction with novel objects over food and enhance social investigation of conspecifics. Novelty-seeking behaviour also likely accounts for classical studies of exploration showing that in the absence of rewards, rats explore and learn the structure of mazes (Tolman, 1948) and prefer to spend more time in unfamiliar maze areas (e.g., Dember, 1956; Hughes, 1968). Maze learning in this context is an example of latent learning, which has parallels to pigeons’ preferences for novelty, unsupervised learning in artificial neural networks, and statistical learning in children (Castro et al., 2018). Some animals also show interest in objects that are not novel but are puzzling or interesting. For example, monkeys engage in solving mechanical puzzle boxes repeatedly over many days in the absence of instrumental rewards (Davis et al., 1950; Harlow, 1950), highlighting that are range of factors that elicit, curiosity-motivated behaviour.

### Non-instrumental choice tasks

**Fig. 1:**
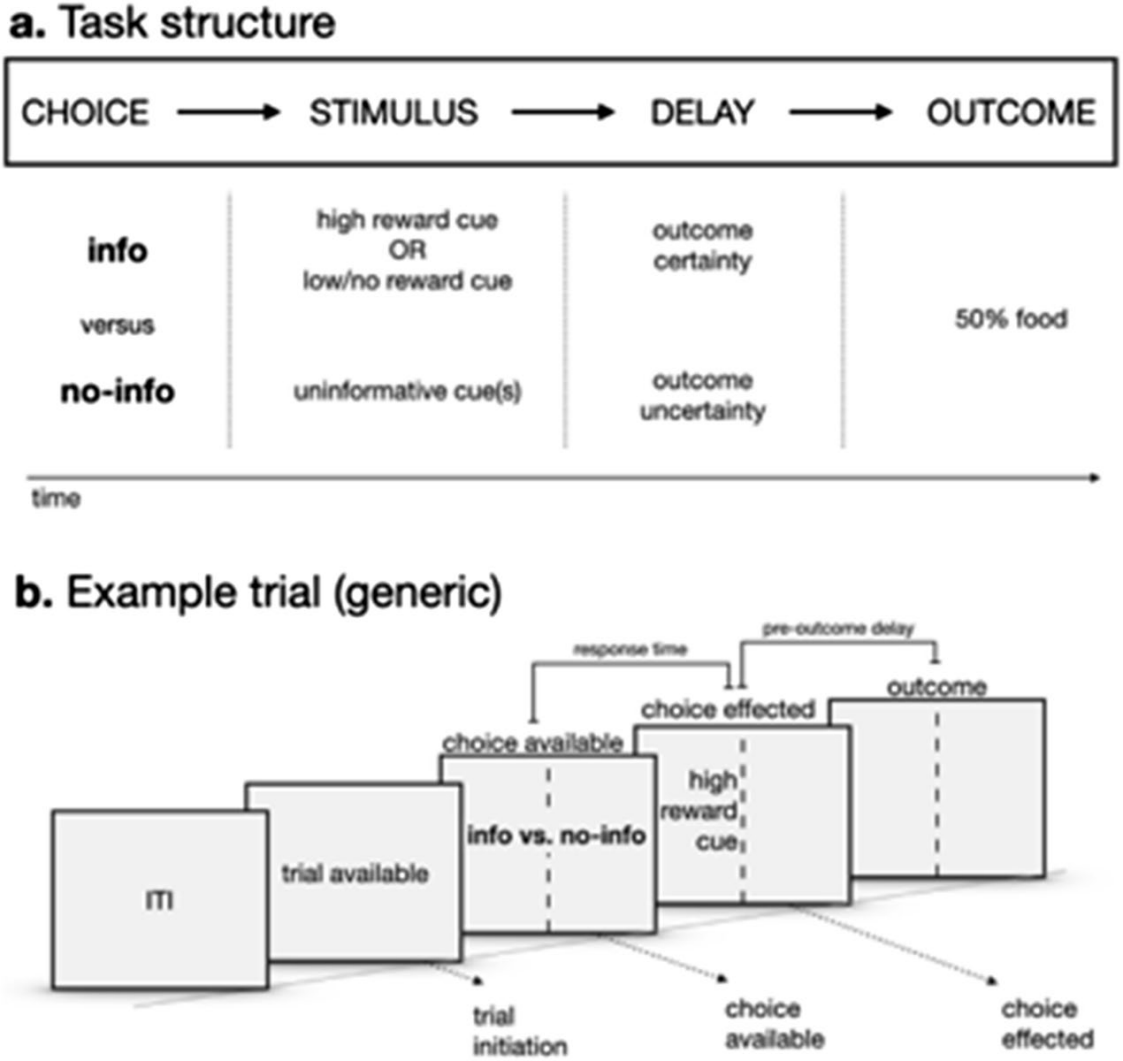
Illustration of non-instrumental choice tasks employed in studies of curiosity and animal cognition. **a.** Task structure. Individuals are offered a choice between two options both of which provide a chance of gaining reward after a short delay period. In the informative option (Info) choice is immediately followed by one of two cues (high reward or low/no reward) which anticipates the outcome due at the end of the delay. The cues in Info provide non-instrumental information because, critically, subjects cannot use the information to alter the reward outcome. In the noninformative option, the cues presented after choice do not signal the trial outcome in advance. **b.** Example trial. choices are separated by an intertrial interval (ITI). Once a trial becomes available subjects can choose between Info and NoInfo. The choice response varies across studies and species, and can include pushing a lever, moving into a choice zone, or pecking a panel. A choice for Info is depicted which then results in the presentation of the high-reward cue, the pre-outcome delay, and then a reward, in this example trial

In recent years researchers aiming to investigate curiosity in both humans and other animals have made empirical and theoretical progress by employing so-called non-instrumental choice paradigms in which individuals can learn to choose alternatives that provide informative cues about probabilistic future outcomes, but cannot then act on that information to increase available rewards (for reviews. see Cervera et al., 2020; Gottlieb & Oudeyer, 2018; Kobayashi & Kable, 2024; Monosov, 2024). In these tasks, which are variably referred to as ‘paradoxical choice’ (e.g., Ojeda et al., 2018) and ‘suboptimal choice’ (e.g., Stagner & Zentall, 2010) in studies of animal cognition, individuals choose between one of two options, which both result in a short delay (e.g., 10 s) and then one of two outcomes: a relatively large reward or no reward (or a smaller reward amount in some studies). One of the options is informative (Info) about trial outcomes, producing reward-predictive signals immediately after the choice response, while the other alternative (NoInfo) does not provide informative signals, so subjects are uncertain about the outcome of a given trial until the delay has lapsed (Fig. 1). Critically, subjects cannot exploit the information in the informative option to improve net rewards in the laboratory—hence, the labelling of the task as ‘non-instrumental’ by some researchers (note, however, that instrumental learning is required for subjects to develop preferences in these tasks). In fact, often subjects must sacrifice available rewards to select Info, because this option frequently provides a lower reward probability/amount than the alternative (e.g., Blanchard et al., 2015; Bussell et al., 2023; Fortes et al., 2016; Ojeda et al., 2018; Vasconcelos et al., 2015).

The non-instrumental choice paradigm is well-suited for contemporary studies of animal curiosity because preferences for the informative option indicate a desire to generate stimuli anticipating future outcomes, though the information cannot be deployed towards obvious extrinsic goals, particularly in cases where the information is costly (Cervera et al., 2020). Moreover, because the task is arbitrarily configured, observed preferences reflect the attribution of value that motivates learned behaviour (rather than innate action sequences), suggesting underlying mechanisms capable of supporting flexible, adaptive behaviour—a hallmark of human curiosity. The relative simplicity of the task means that it has the potential to be implemented across a range of species, while the fundamental task structure is preserved. Nonetheless, the extent to which preferences in non-instrumental tasks reflects curiosity in animals as opposed to other motivations is actively debated (e.g., Ajuwon et al. 2023; Vasconcelos et al. 2015).

Though recently adopted to explore curiosity, the origin of non-instrumental choice tasks can be traced back almost 75 years. At the time, experimental psychologists discovered that pigeons and rats learned to perform actions—labelled ‘observing responses’—to generate outcome-predictive stimuli that did not affect reward rate (see Dinsmoor, 1983; Prokasy, 1956; Wyckoff, 1969). The original observing response studies have led to extensive, ongoing research in experimental psychology aimed at charactering experimental factors involved in the observing response, as well as preferences in tasks derived from it (e.g., Anselme & Blaisdell, 2024; Zentall et al., 2019a). Pigeons have proved an excellent model to investigate cognitive mechanisms and associate processes underpinning robust preferences that are not motivated by primary rewards (for reviews see Cunningham & Shahan, 2018; Dunn et al., 2023; González et al., 2023; Zentall, 2016).

**Fig. 2:**
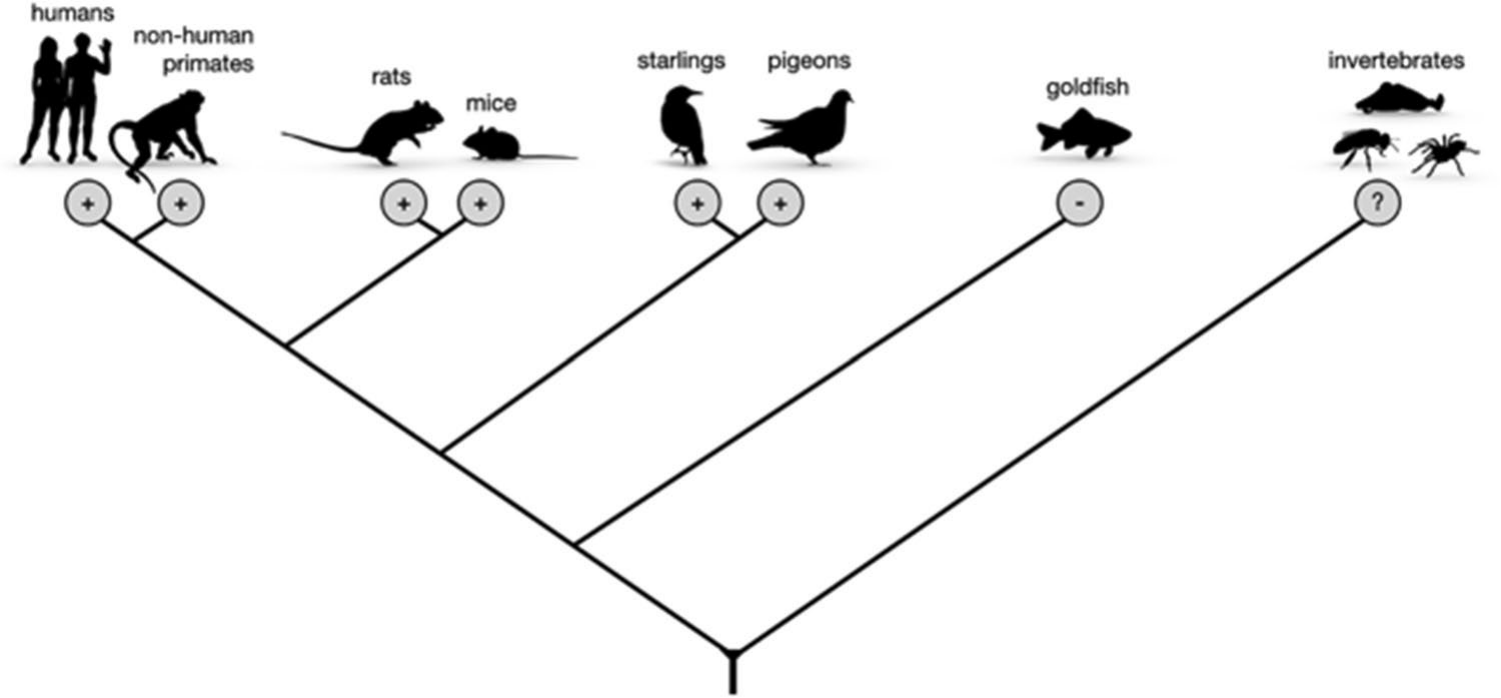
Preference in non-instrumental (paradoxical) choice tasks across species. The simplified cladogram depicts preferences for the option providing non-instrumental information across tested species. Plus signs indicate preference for the informative option while the minus sign depicts lack of evidence of preference. Invertebrates are depicted as a single group here to highlight that no data is currently available for any invertebrate species. All animal silhouettes from are from https://www.phylopic.org/

The key finding from non-instrumental tasks across neuroscience, cognitive psychology, experimental psychology and behavioural ecology is that a range of mammalian and avian species, including humans (Bennett et al., 2016; Bode et al., 2023; Bromberg-Martin et al., 2024; Brydevall et al., 2018; Iigaya et al., 2016; Kobayashi et al., 2019), monkeys (Blanchard et al., 2015; Bromberg-Martin & Hikosaka, 2009, 2011), rats (Ajuwon et al., 2023; Cunningham & Shahan, 2019; Ojeda et al., 2018), mice (Bussell et al., 2023), pigeons (Fortes et al., 2018 ; González et al., 2023; McDevitt et al., 2022, 2024; Smith & Zentall, 2016; Zentall et al., 2022), and starlings (Vasconcelos et al., 2015) show robust preferences for the informative option (Fig. 2). Remarkably, pigeons and starlings prefer the informative option even when it is five times less profitable than the alternative. These observations are striking because they challenge traditional thinking about the function of decision-making mechanisms that operate in animal brains (Stephens & Krebs, 1986) and have therefore been labelled ‘paradoxical’ and even ‘suboptimal’. Though empirically well established, the interpretation of preferences in non-instrumental tasks across animals is under active debate and has facilitated interdisciplinary, theoretical advances in the study of cognition and intelligence. Below, we outline several accounts of these intriguing preferences, highlighting evidence across species.

## Does curiosity underly preference in non-instrumental tasks?

### Information as reward

One interpretation of results from non-instrumental paradigms suggests that preference is primarily driven by information-seeking that is an analogue (or homologue) to human curiosity—an intrinsic motivation to acquire knowledge—and mediated by mechanisms aimed at resolving uncertainty (Cervera et al., 2020; Gottlieb & Oudeyer, 2018; Monosov, 2024). This interpretation proposes that information can act as a primary reinforcer in learning processes (Berlyne, 1957, 1960; Hendry, 1969). Supporting this view, research in both humans (Bromberg-Martin et al., 2024; Brydevall et al., 2018; Charpentier et al., 2018) and macaques (Bromberg-Martin et al., 2024; Bromberg-Martin & Hikosaka, 2009, 2011) indicates that midbrain dopamine neurons, which are known to represent the value of primary rewards, such as food and water, also encode the subjective value of information. Additionally, individual neurons in mammalian neocortex represent information value (Blanchard et al., 2015; Bussell et al., 2023; White et al., 2019). Both humans and monkeys use similar computations to quantify uncertainty and demonstrate increased preferences for information when uncertainty is higher (Bromberg-Martin et al., 2024; Bromberg-Martin & Hikosaka, 2011). Furthermore, like humans (Rodriguez Cabrero et al., 2019), monkeys also value information more when it is delivered earlier rather than later (Bromberg-Martin et al., 2024; Bromberg-Martin & Hikosaka, 2011), a result also mirrored in starlings (Vasconcelos et al., 2015). This is consistent with the view that extended periods of uncertainty are aversive (Bennett et al., 2016), with information gain acting as a negative reinforcer. It is also likely that information gain, or uncertainty resolution, acts as a positive reinforcer, where information is itself appetitive, as this would motivate individuals to actively explore and build a better world model, a notion consistent with active inference theory (Friston et al., 2017), preferences for counterfactual information in macaques (Wang & Hayden, 2019) and dopaminergic bonuses elicited by reward predictive stimuli (e.g., Bromberg-Martin & Hikosaka, 2009).

However, clear support for a reinforcing effect of uncertainty reduction, and thus curiosity-like preferences across all tested species is not yet conclusive. For example, while González et al. (2020) observed that pigeons’ preferences for the informative option increase with outcome uncertainty, Roper and Zentall (1999) did not observe this effect, and further investigations in a broader array of species are necessary. Moreover, if reducing uncertainty reduction is the main motivator of preference, individuals should be interested in gaining informative stimuli irrespective of their valence—whether they predict ‘good’ or ‘bad’ outcomes. While findings in rats (Ajuwon et al., 2023) demonstrate a preference for Info even in the absence of a salient stimulus announcing forthcoming rewards after choice, pigeons do not appear motivated to reduce uncertainty when only adverse outcomes are clearly indicated (Dinsmoor et al., 1972; Jenkins & Boakes, 1973; Kendall, 1973; Silberberg & Fantino, 2010; for reviews, see Dinsmoor, 1983; Shahan & Cunningham, 2015).

### Conditioned reinforcement from good news

Another interpretation of preferences in non-instrumental tasks, which does not necessarily depend on individuals being motivated by reductions in uncertainty, focuses on the asymmetrical conditioning of stimuli associated with reward outcomes. This associative view suggests that preferences in non-instrumental tasks arise because stimuli paired with positive outcomes become appetitive, conditioned reinforcers. These reinforces promote choices that generate those stimuli, while stimuli paired with negative outcomes (such as omission of food delivery) are weakly inhibitory or ignored all together (Bower et al., 1966; Prokasy, 1956; Wyckoff, 1959). In essence, subjects opt for the informative choice as they seek to generate the ‘good news’ stimulus strongly associated with reward, but they show less avoidance towards the ‘bad news’ presented during the trials with little/no reward. This account is compelling as it does not need to rely on the evolution of sensitivity to abstract concepts such as uncertainty. Nonetheless, among the numerous models of conditioned reinforcement in paradoxical choice (e.g., Beierholm & Dayan, 2010; Case & Zentall, 2018; Gipson et al., 2009; Zentall, 2013; comprehensibly reviewed in González et al., 2023), some have argued that uncertainty reduction might play a role in giving secondary reinforcing properties to good news stimuli (see Cunningham & Shahan, 2018; Dunn et al., 2023). These models, though, diverge from the broader idea that uncertainty reduction itself is reinforcing, as they indicate that stimuli announcing negative outcomes are not actively sought and focus mainly on stimuli associated with food rewards, rather than broader information-seeking.

The models incorporating uncertainty reduction into conditioned reinforcement (Cunningham & Shahan, 2018; Dunn et al., 2023) have effectively explained data from non-instrumental tasks in pigeons, and are supported by evidence that generally, across species, the stimulus for ‘good news’ in Info has a greater impact on preference than the stimuli associated with less reward (Ajuwon et al., 2023; Fortes et al., 2017; Laude et al., 2014; McDevitt et al., 1997; Pisklak et al., 2015; Spetch et al., 1994). A recent study exploring paradoxical choice in goldfish, however, suggests a different story. The authors argued that the lack of preference for advanced information they observed raised the possibility that widespread conditioning mechanisms across species are not always sufficient to motivate Info preference, supporting the view that specialised mechanisms aimed at reducing uncertainty have evolved in those species that do show the preference (Ajuwon et al., 2024). It is also worth noting that the drive to reduce uncertainty and generate conditioned reinforcers need not be mutually exclusive mechanisms—data in rats (Ajuwon et al., 2023) and monkeys (Daddaoua et al., 2016) are consistent with the possibility that both mechanisms can operate simultaneously during decision-making.

The idea that good news stimuli play the critical role in promoting preference in non-instrumental tasks is also reflected in the contention that savouring of positive future outcomes motivates preferences (Iigaya et al., 2016, 2020; Kobayashi et al., 2019). This view, developed from human studies, takes a reinforcement learning approach and is informed by the concept of anticipatory utility in economics (Grant et al., 1998; Kreps & Porteus, 1978). Like some proponents of the conditioned reinforcement account (Zentall, 2023; Zentall et al., 2019b; Zentall & Stagner, 2011), Iigaya et al. (2016, 2020) argue that preferences in non-instrumental tasks may reflect potentially maladaptive mechanisms that are implicated in pathological gambling behaviour.

### Extrinsic urges

The interpretations of apparent information-seeking behaviour so far have focused on mechanisms that drive behaviour in real time, with less focus on the adaptive function of preferences for non-instrumental information observed in the laboratory. In a study on starlings, Vasconcelos et al. (2015) aimed to address this gap by developing a model to explain preferences based on optimal foraging theory. They argued that preferences for advanced non-instrumental information in the lab actually reflect adaptive mechanisms that are rate maximising in the natural environment of evolutionary adaptation, where information about forthcoming rewards will likely be useable. The authors argued that the paradoxical preferences in the lab result from subjects being placed in an artificial situation where information about forthcoming reward is provided but non-instrumental, causing the misfiring of otherwise adaptive mechanisms (Vasconcelos et al., 2018). The model predicts strong preference for Info, even when doing so is submaximal in the lab because when presented with a stimulus for no reward in Info, subjects disengage from the task. The implication of this perspective is that rather than reflecting subjective information value per se, preferences in paradoxical choice are generated by mechanisms aimed at maximising extrinsic rewards, and therefore need not reflect curiosity. In its original formulation, this model could not account for the finding that unlike pigeons and starlings, rats do not continue to show preference for Info when it is very costly to do so, though a number of pigeon studies have corroborated the models’ predictions and extended it to account for interspecies differences in preference (Fortes et al., 2016, 2017, 2018).

A related argument referred to as the preparation hypothesis suggests that preference in non-instrumental tasks can be explained by the fact that while individuals cannot use the outcome predictive signals in Info to improve reward rate, they can use these stimuli to improve the usefulness of outcomes when they do arrive, by preparing for them (e.g., by salivating or moving towards the site of reward delivery earlier; Mackintosh, 1974). It is also possible that individuals can save time and energy on unrewarded trials by doing the opposite in the informative option. This is an important possibility to consider, but it becomes less likely to account for preferences when selecting the informative option is very costly, and the putative extrinsic benefits of anticipation seem unlikely to outweigh significant losses individuals are willing to incur.

### The story so far

**Fig. 3:**
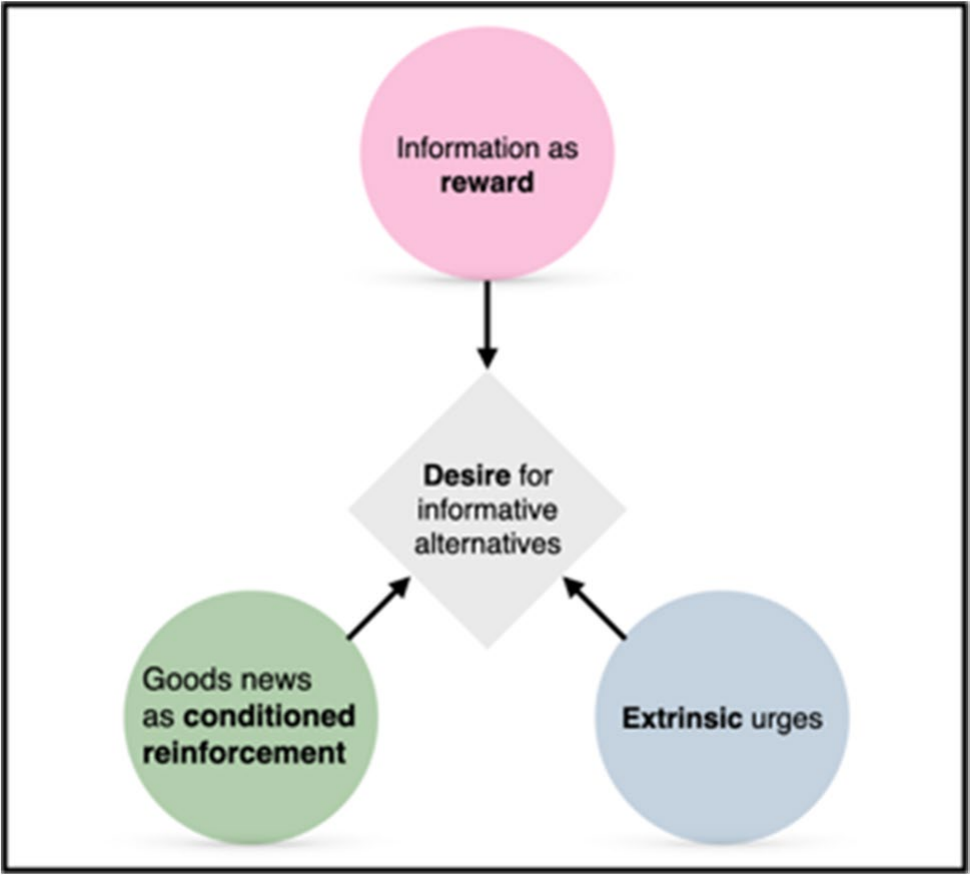
Proposed interpretations of preferences for options that offer information about future outcomes in non-instrumental tasks. Information gain may be intrinsically reinforcing—consistent with the notion of curiosity. Preferences may also arise from secondary conditioning of signals for rewards specifically, as opposed to a broader drive to seek non-instrumental information. Furthermore, preferences could be caused by mechanisms evolved to deal with instrumental information in natural circumstances. The primacy of these varied interpretations is actively debated, but they are not mutually exclusive

To summarise, a range of mammalian and avian species show robust preferences for alternatives providing non-instrumental information. This finding has fostered interdisciplinary, advances aimed at understanding the underlying functions and mechanisms that drive this intriguing behaviour (Fig. 3). Studies involving humans and macaques provide support for the idea that individuals are driven by an intrinsic motivation to reduce uncertainty—curiosity, or at least a rudimentary form of it. However, there is also evidence supporting other interpretations that are not necessarily mutually exclusive. Secondary conditioning motivates individuals to generate appetitive ‘good news’ stimuli in non-instrumental tasks, and individuals may seek these stimuli to savour future outcomes—mechanisms that have been suggested as factors promoting maladaptive gambling behaviour. Another possibility is that preferences are driven by mechanisms evolved to deal with instrumental information and reward maximisation during foraging, mechanisms which inadvertently misfire and shape preference in non-instrumental tasks in the lab. Comparative trends have also started to emerge, with evidence suggesting that birds are less sensitive to reward loss than mammals, and one species of fish displaying no preference in choices for advanced information (despite having learnt task contingencies), raising functional questions about species differences. These increasingly interdisciplinary efforts emphasise the utility of non-instrumental tasks and highlight that decision-making theories ought to account for the subjective and intrinsic value that individuals attribute to informative stimuli, a previously neglected but increasingly appreciated feature of animal behaviour and cognition. In the next section, we highlight open questions and discuss new avenues for future research.

## Looking forward

### The question of animal curiosity

The finding that individuals are willing to sacrifice rewards for stimuli that anticipate future outcomes but are instrumentally redundant, appears to reflect curiosity. Nonetheless, data from non-instrumental tasks support alternative interpretations (e.g., Anselme 2023), and the most convincing evidence for human-like curiosity comes primarily from one species, the rhesus macaque. The extent to which non-human animals generally show flexible forms of curiosity aimed at reducing uncertainty therefore remains an open question. To build more convincing evidence of curiosity-like behaviour in animals, non-instrumental tasks could explore whether a wider range of animals seek information about a broader set of outcomes. In most non-instrumental experiments, individuals can seek information about the magnitude or timing of upcoming food rewards (though see Lockard, 1963, for rats seeking information about upcoming shocks). However, genuinely curious individuals would be expected to seek varied kinds of information about reward (e.g., quality, type), as well as a diverse set of outcomes than are unrelated to consumable rewards, including counterfactual information. Future studies could explore whether participants seek social information about other individuals, aversive but not harmful events such as sleep deprivation, or the structure of their habitat.

A more general issue is the current lack of consensus around a holistic, widely applicable definition of curiosity (see Forss et al., 2024, for a range of perspectives). In studies on adult humans, it is a forgone conclusion that experimental subjects are acting out of curiosity, because in many cases they are able to communicate their subjective experience of how curious they feel via self-report. Without this ability to know the internal experiences of animals, how can we be sure experimental subjects are acting out of curiosity in laboratory tasks? To tackle this issue, it is instructive to consider another line of research within comparative cognition, where researchers faced a similar problem. In that case, the question was whether nonhuman animals have episodic memory (Tulving, 1983), the ability that allows humans to revisit past events in their minds eye, and ‘mentally travel in time’. Then, episodic memory was considered uniquely human (Suddendorf & Corballis, 1997), and definitions were based in terms of conscious experiences of time, ‘chronesthesia’ and self, ‘autonoesis’ (Tulving, 2002), making it difficult to examine the prospect of episodic memory in nonhuman animals. To resolve this issue, Clayton and Dickinson (1998) focused on the key behavioural feature of human episodic memory that could be experimentally explored in a nonhuman animal, the ability to retrieve information about ‘where’ a unique event or ‘episode’ took place, ‘what’ occurred during the event and ‘when’ it happened (Tulving, 1972). Clayton and Dickinson (1998) leveraged food-caching behaviour in scrub jays, finding that individuals could form unified and integrated memories about single caching events, remembering ‘what’ items they had cached, ‘where’ they had stored them, and ‘when’ it had been. Because they did not address phenomenological factors, Clayton and Dickinson (1998) termed this discovery episodic-like memory (Griffiths et al., 1999). This landmark study encouraged further development and expansion of the behavioural criteria for episodic-like memory, including its content, structure, and flexibility (Clayton et al., 2003), enabling converging lines of evidence across taxa to help establish the prevalence of episodic-like memory in nonhuman animals. This approach of developing clearly defined behavioural criteria that are amenable to laboratory testing (and revision) was fruitful in the case of episodic-memory research. In that vein, below we outline some behavioural features of human curiosity that could form behavioural criteria for curiosity-like behaviour in animals, which may offer useful avenues for future research.

### Features of curiosity

An emerging view in cognitive psychology proposes that curiosity functions not only to reduce environmental uncertainty but also to maximise learning progress, enabling agents to build a more accurate world model (Dubey & Griffiths, 2020; Modirshanechi et al., 2023; Poli et al., 2024; see also Wang & Hayden, 2021, suggesting curiosity helps agents build a cognitive map of the environment). This view is inspired by research in curiosity-driven artificial intelligence and robotics, where algorithms have been developed enabling artificial agents to autonomously generate their own learning goals (Kaplan & Oudeyer, 2007a, b). Research on infant attention (Poli et al., 2020) has found that participants spend more time looking at sequences of stimuli that maximise their learning rate about the prospective location of future stimuli. Moreover, adults participating in online games self-organise their exploration of these games in a manner that maximises learning progress (Ten et al., 2021). Future studies of animal curiosity could explore the learning progress hypothesis, and the role of metacognition in curiosity (see Wade & Kidd, 2019) more generally, which would be complemented by the development of more open-ended and ecologically relevant experimental paradigms in animal curiosity research (Hunt et al., 2021). The use of more open-ended non-instrumental paradigms would also help facilitate research into the links between non-instrumental information-seeking in choice tasks, and embodied exploration in unmapped environments—another important feature of curiosity.

**Fig. 4:**
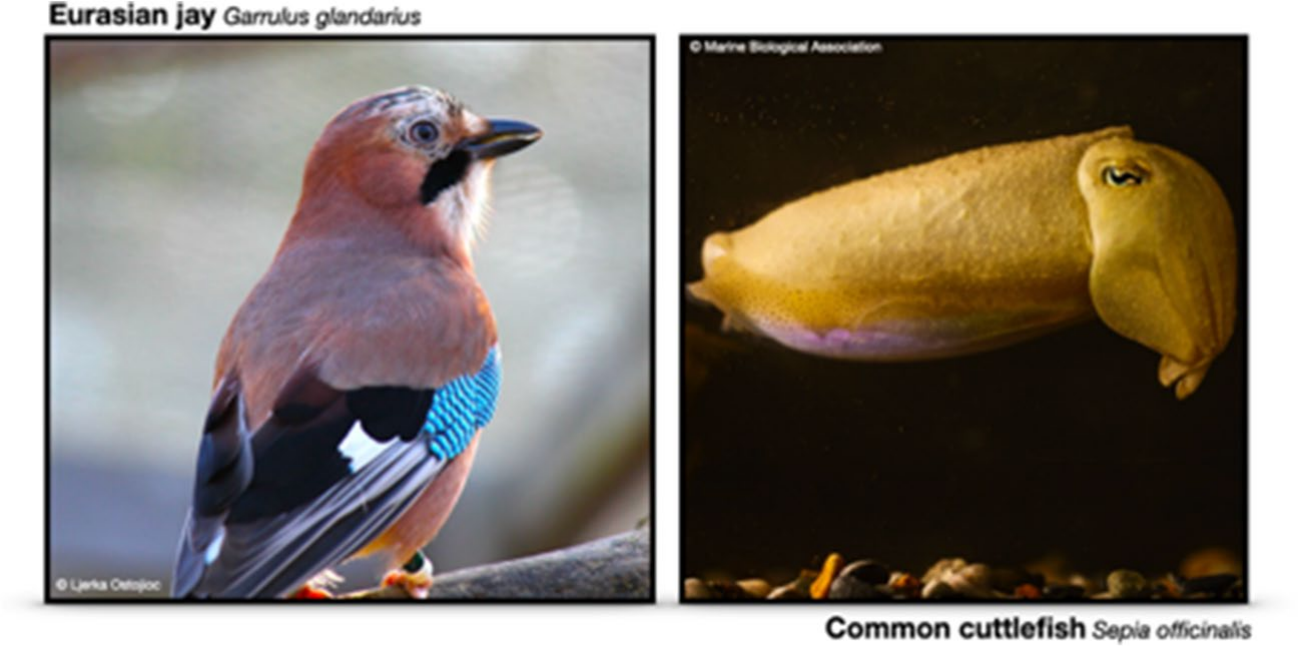
Food-caching birds such as the Eurasian jay (left) and cephalopods like the common cuttlefish (right) could serve as useful models for future research exploring the cognitive and evolutionary underpinnings of curiosity-like behaviour across animals

If curiosity serves to build an accurate world model, then it could be said to function ‘with a future in mind’ (Suddendorf & Busby, 2005). One reason that curiosity is so useful is that it motivates individuals to learn about their environment, even when the utility of information at the time of acquisition is computationally intractable, allowing individuals to benefit from that information over the course of extended periods. In non-instrumental tasks, information is provided about outcomes that are very close to the time of choosing (typically <15 s), but our human curiosity leads us to seek information about events that may occur far into the future, and we are able to remember information long after we sought it, sometimes through episodic recall. In addition to reducing uncertainty about particular events, and maximising learning progress, curiosity may therefore function to enrich declarative memory (a flexible form of long-term memory which includes episodic recall; Squire et al., 1993). This is consistent with the finding that the people are better able to remember the answers to questions that piqued their curiosity (e.g., Gruber et al., 2014; Kang et al., 2009). It would therefore be useful to examine whether nonhuman animals can spontaneously deploy previously sought non-instrumental information, even after extended periods of time. Food-caching birds could serve as a useful model to explore this. Eurasian jays (Fig. 4) are able to incidentally encode aspects of their environment and recall this information more than 24 hours after they acquired it (Davies et al., 2024). Furthermore, scrub jays are able to plan for the future without reference to their current motivational state (Raby et al., 2007), indicating a strong selection pressure to recall information gained in the past, for use in the future.

Another feature of human curiosity is that the intensity of our curiosity varies over time and is affected by a range of intrinsic and extrinsic factors. In their Prediction, Appraisal, Curiosity and Exploration (PACE) framework, Gruber and Rangnath (2019) highlight that prediction errors arising from unexpected context changes, or violations of expectation, may enhance our state of curiosity and increase information-seeking (e.g., Hannula et al., 2012; Risko et al., 2012). Future work exploring curiosity in animals could investigate whether there are factors that alter subjects’ desire for advanced information, particularly whether context changes or violations of expectation induce heightened information-seeking. Violations of expectation have recently been observed in Eurasian jays, suggesting they could be a useful model to explore these factors. Jays were presented with a magic effect in order to identify blind spots and roadblocks in cognitive processing which arise from primed causal expectations (Garcia-Pelegrin et al., 2020, 2024). Schnell et al. (2021d), adapted the ‘cups-and-balls’ magic routine, where some birds had occluded food items swapped with unexpected rewards. They found that jays spent more time inspecting cups when their expectations were violated and took longer to consume rewards that had been swapped in violation of their expectation, especially if the outcome was less desired than the one expected. These results highlight the potential utility of magic-based paradigms as a tool to elicit violations of expectation and explore effects on information-seeking and curiosity.

### Evolution and development

Over half a century ago, the Nobel Prize–winning ethologist Niko Tinbergen opined that in order to comprehensively understand behaviour, we need to consider four key factors: the underling proximate mechanisms, development over the life span, behaviours’ adaptive function, and the underlying evolutionary history (Tinbergen, 1963). Within Tinbergen’s framework, two important aspects of behaviour in non-instrumental tasks have been relatively neglected: ontogeny and evolutionary history. So far, non-instrumental tasks have been restricted to a relatively small number of vertebrate species (Fig. 2), and virtually nothing is known about the ontogeny of preference for the informative option. Studying both of these elements of behaviour comes with challenges: to study ontogeny the contingencies of subjects differing in developmental stages needs to remain similar over time, while studying evolutionary history requires novel behavioural models from unexplored taxa.

We suggest that the common cuttlefish (*Sepia officinalis*; Fig. 4), an emerging model in cognitive research, is ideal for tackling questions pertaining to the evolutionary history and development of information-seeking behaviour that may reflect curiosity. Cuttlefish are highly motivated by food rewards, show future-oriented decision-making (Billard et al., 2020; Schnell et al., 2021b), and have excellent visual-spatial cognitive skills (Scatà et al., 2016), including episodic-like memories (Jozet-Alves et al., 2013), indicating that they would be able to quickly learn the contingencies in non-instrumental tasks. The lineage to which cuttlefish belong, the coleoid cephalopod molluscs, diverged from vertebrates 500 million years ago, and likely independently evolved sophisticated cognition under different socioecological conditions to traditional vertebrate models (Amodio et al., 2019; Schnell et al., 2021a). The discovery of a drive for advanced non-instrumental information in this species would clarify whether this trait has evolved independently more than once, and would suggest that the underlying mechanisms are adaptive, important components of sophisticated cognition. This would also elucidate whether divergent socio-ecological selection pressures and neural architectures can give rise to subjective information value.

Moreover, cuttlefish are relatively short lived (around 2 years), making them an ideal model to investigate the development of traits across the entire life span (e.g., Schnell et al., 2021c). Investigating the ontogeny of paradoxical choice in cuttlefish may also provide functional insights. If preference in non-instrumental tasks reflects human-like curiosity, then we may expect that information-seeking behaviour functions to enable younger individuals to acquire knowledge that is useful for solving novel problems in the future (Kidd & Hayden, 2015). Examining developmental changes could corroborate this proposal if younger individuals show a stronger preference for information in the paradoxical choice task information than older, more experienced, individuals.

## Conclusion

Over the past few decades, research leveraging non-instrumental choice paradigms has demonstrated that a range of mammalian and avian species, including humans, have robust preferences for informative cues anticipating future outcomes, even though the information lacks instrumental utility and often comes at the cost of primary rewards. The finding that individuals attribute subjective value to prospective information has propelled interdisciplinary advances in our understanding of decision-making mechanisms that operate in the brain, while highlighting that curiosity may influence decision-making in a range of nonhuman animals.

Curiosity is a complex motivator of behaviour that arguably has been critical to the evolutionary success of *Homo* *sapiens*, and potentially plays an important role in the decision-making of other species. Nonetheless our understanding of the evolutionary origins of curiosity is at its infancy. Presently, evidence from primate research offers the strongest support for the notion that curiosity influences behaviour during decision-making in nonhuman animals. However, the extent to which curiosity influences decision-making across taxa, particularly in invertebrates, remains unclear. Continued efforts to explore candidates for curiosity-motivated behaviour in a range of taxa should help to refine theoretical notions of curiosity and open avenues to investigate the cognitive principles and evolutionary pressures that support curiosity-driven behaviour.

Highlighting several features of human curiosity, we propose directions for future research aimed at characterising and understanding curiosity in nonhuman animals. In our current ‘disinformation age’ (Bennett & Livingston, 2020), addressing such questions is increasingly critical. Doing so will deepen our understanding of how natural selection has shaped decision-making mechanisms, offering insights into human mental health (Bennett et al., 2021; Charpentier et al., 2022), the idiosyncrasies of human cognition, and the nature and evolution of intelligence.

## Data Availability

Not applicable.
