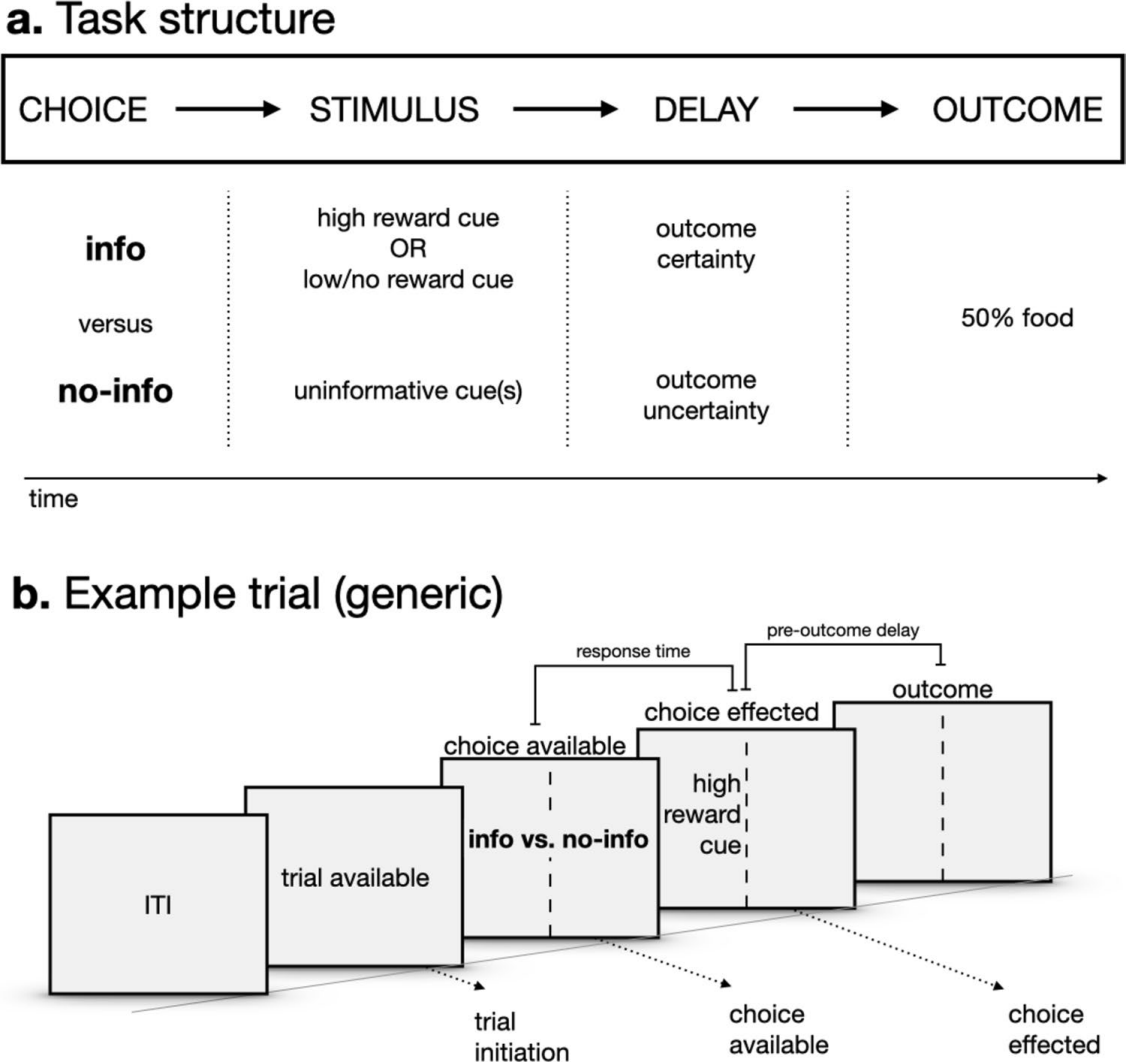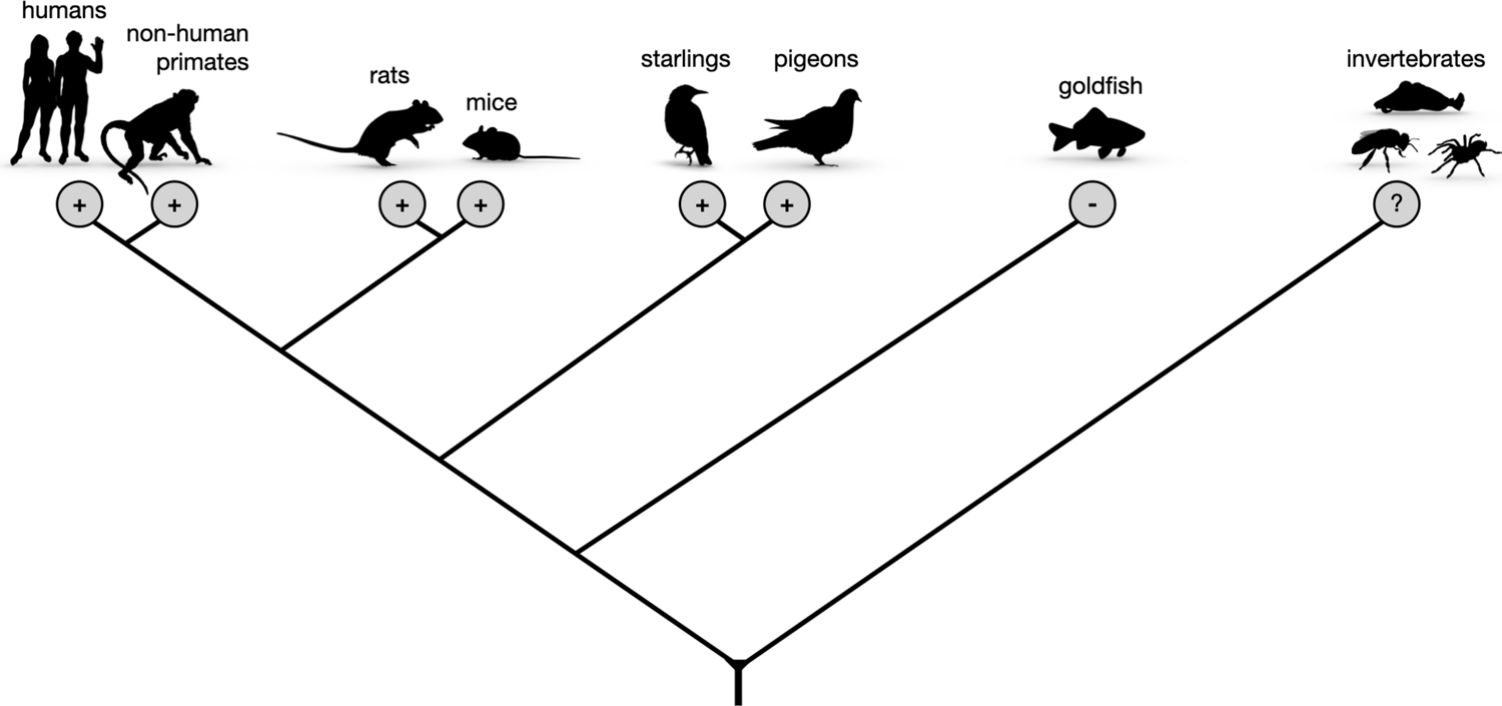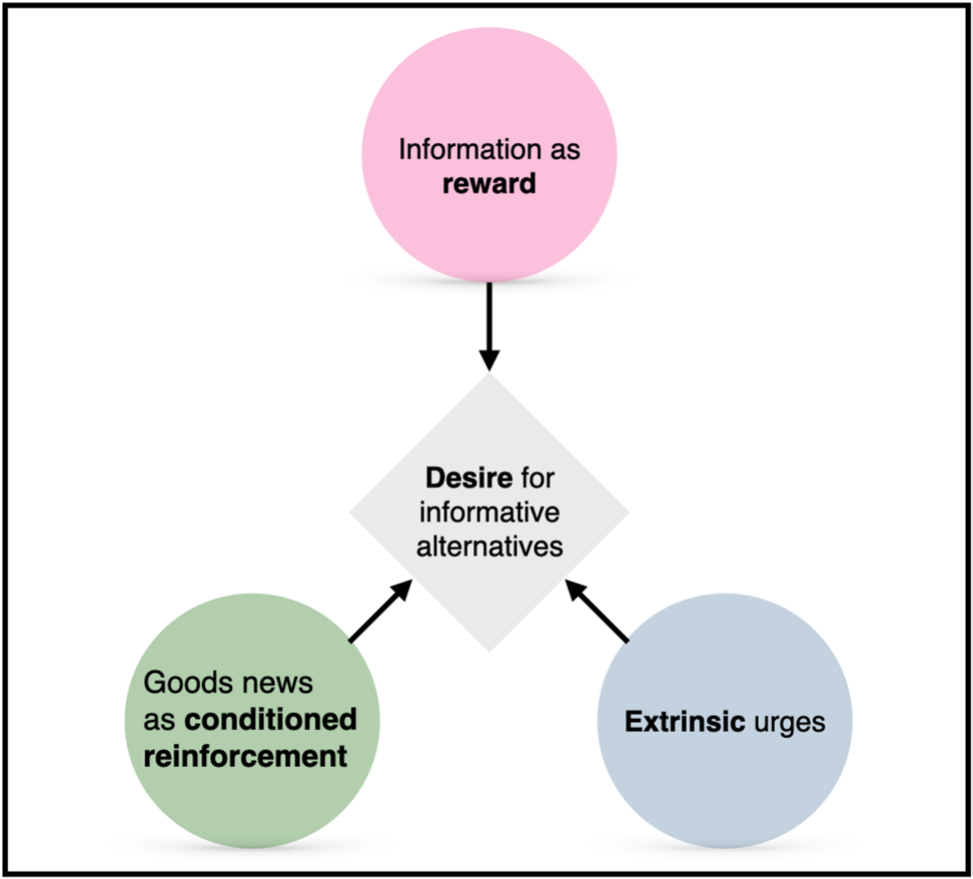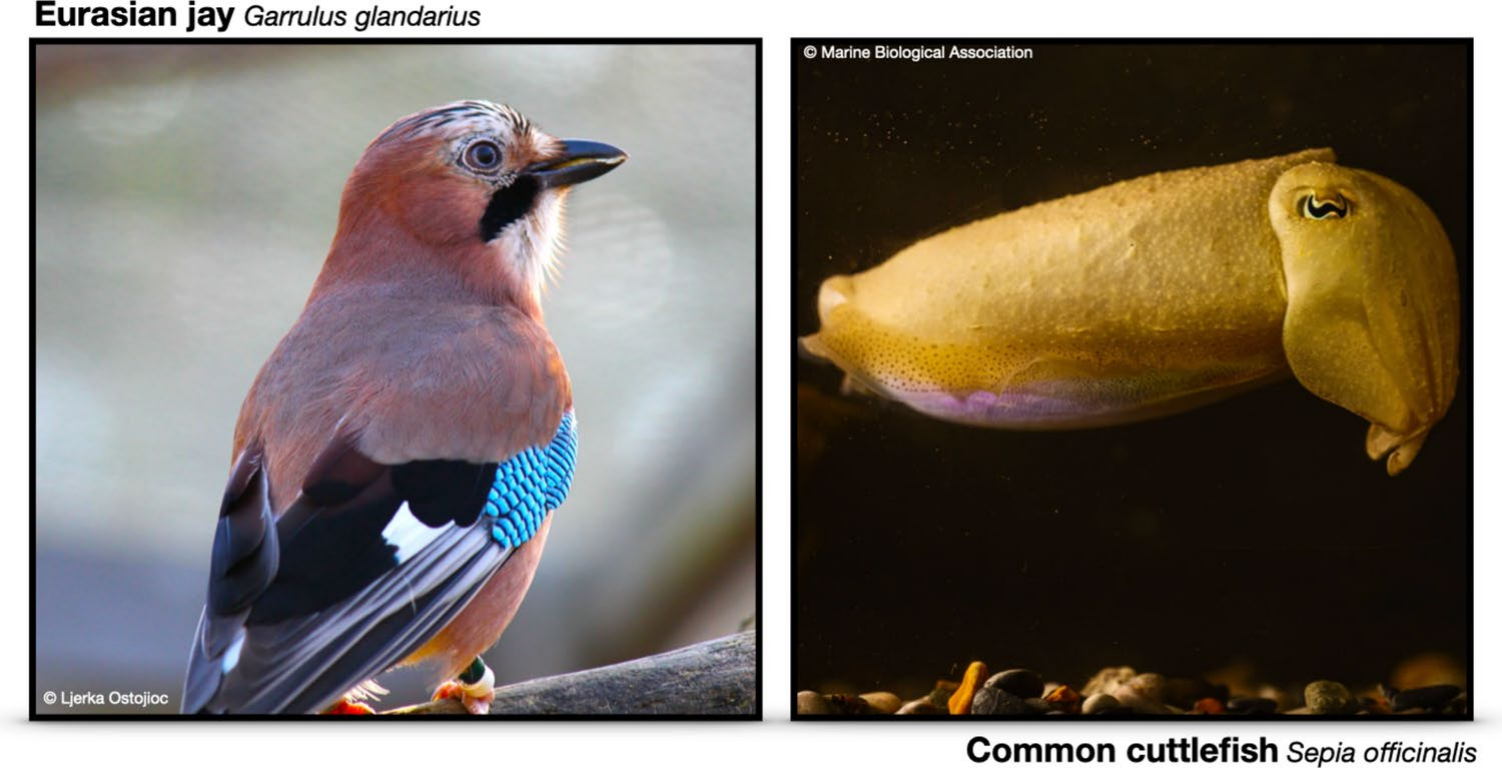# Correction: To know or not to know? Curiosity and the value of prospective information in animals

**DOI:** 10.3758/s13420-025-00667-2

**Published:** 2025-03-13

**Authors:** Victor Ajuwon, Tiago Monteiro, Alexandra K. Schnell, Nicola S. Clayton

**Affiliations:** 1 Department of Psychology, University of Cambridge, Cambridge, UK; 2 William James Centre for Research, University of Aveiro, Aveiro, Portugal; 3 Domestication Lab, Department of Interdisciplinary Life Sciences, Konrad Lorenz Institute of Ethology, University of Veterinary Medicine Vienna, Vienna, Austria

**Correction: Learning & Behavior**

10.3758/s13420-024-00647-y

The paper included lower resolution figures. Higher resolution figures are presented here.